# Left Bundle Branch Block as a Risk Factor for Heart Failure

**DOI:** 10.1001/jamanetworkopen.2025.25801

**Published:** 2025-08-07

**Authors:** Anna-Sophie Thein, Shalini Dixit, Elsayed Z. Soliman, Susan R. Heckbert, Bruce M. Psaty, John Gottdiener, Gregory M. Marcus

**Affiliations:** 1 Division of Cardiology, Department of Medicine, University of California, San Francisco; 2 Department of Drug Design and Pharmacology, University of Copenhagen, Copenhagen, Denmark; 3 Epidemiological Cardiology Research Center, Section on Cardiovascular Medicine, Wake Forest University School of Medicine, Winston Salem, North Carolina; 4 Department of Epidemiology, School of Public Health, University of Washington, Seattle; 5 Cardiovascular Health Research Unit, Department of Medicine, University of Washington, Seattle; 6 Department of Medicine, Division of Cardiology, University of Maryland School of Medicine, Baltimore; 7 Department of Health Systems and Population Health, University of Washington, Seattle

## Abstract

**Importance:**

Left bundle branch block (LBBB) might play a causative role in the progression to heart failure (HF), but the association of LBBB with HF in asymptomatic individuals with structurally normal hearts has not yet been investigated.

**Objectives:**

To examine the association of LBBB with the risk of HF in community-dwelling individuals.

**Design, Setting, and Participants:**

This longitudinal cohort study used data from 4 US sites participating in the Cardiovascular Health Study (1989-1990), collected from 1989 to 2013. Adults aged 65 years and older with a normal left ventricular ejection fraction (LVEF) and no history of HF at baseline were included. Data were analyzed from February 2018 to October 2024.

**Exposure:**

The presence of LBBB as confirmed by 12-lead electrocardiograms obtained on all participants at baseline.

**Main Outcomes and Measures:**

Multivariable Cox proportional hazards models were used to estimate the associations of LBBB with the risk of incident HF, hospital admission for HF with reduced EF or HF with preserved EF, 5-year LVEF decline, and death.

**Results:**

Among 4541 individuals (mean [SD] age, 72.6 [5.5] years; 2697 female [59.4%]), 44 (1.0%) exhibited LBBB at baseline, and 1321 (29.1%) received a diagnosis of HF over a median (IQR) follow-up of 14.6 (8.4-18.3) years. In the adjusted model, those with baseline LBBB had an increased risk of incident HF (hazard ratio, 4.98; 95% CI, 2.18-11.39; *P* < .001) and greater odds of 5-year LVEF decline (odds ratio, 4.73; 95% CI, 1.70-13.70; *P* = .003), but no statistically significant increased risk of death was observed (hazard ratio, 1.39; 95% CI, 0.99-1.94; *P* = .05).

**Conclusions and Relevance:**

In this cohort study of individuals with structurally normal hearts, LBBB was associated with an increased risk of HF and a decrease in LVEF, suggesting that LBBB could be a potential target for early interventions to prevent deterioration of cardiac function and, ultimately, progression to HF.

## Key Points

**Question:**

Is left bundle branch block in asymptomatic individuals with structurally normal hearts associated with an increased risk of heart failure, decline in left ventricular function, or death?

**Findings:**

In this cohort study of 4541 individuals from 4 US sites, those with baseline left bundle branch block had a 5-fold increased risk of incident heart failure and 4-fold greater odds of left ventricular decline after 5 years.

**Meaning:**

These findings suggest that left bundle branch block might play a causative role in the progression to heart failure and may serve as an important marker for surveillance protocols and early preventive interventions.

## Teaser

This cohort study analyzes associations of left bundle branch block with heart failure among adults aged 65 years or older from the Cardiovascular Health Study.

## Introduction

Left bundle branch block (LBBB) is associated with increased mortality in patients with heart failure (HF) and reduced left ventricular ejection fraction (LVEF).^1,2,3,4^ Cardiac resynchronization therapy is a well-established treatment to mitigate the morbidity and mortality of LBBB in these patients.^5,6,7^ However, incidental LBBB is sometimes detected in asymptomatic individuals, and previous studies suggest an association of LBBB in patients without HF with the risk of developing HF and dying from cardiovascular disease.^8,9,10^ However, the consequences of LBBB restricted to patients in the general population with structurally normal hearts, as confirmed with an image-based approach, have not yet been investigated, and it remains unclear if LBBB reflects the progression of undiagnosed underlying cardiac disease or if it is an independent risk factor.^11,12,13,14^ Although current knowledge is limited, studies allude to a potential causative role of LBBB in the progression to HF through mechanical dyssynchrony, leading to increased LV wall stress, asymmetric hypertrophy, and dilatation.^12,15,16^ We therefore sought to examine the association of LBBB in community-dwelling adults with the risk of HF development and death. Because there are no current recommendations for further testing or treating patients with asymptomatic LBBB, improving our understanding of these associations may help inform guidelines, identify individuals at high risk of progression to HF, and, ultimately, help uncover novel effective interventions.

## Methods

This cohort study received a certificate of approval to conduct these analyses of deidentified data from the University of California institutional review board. The study followed the Strengthening the Reporting of Observational Studies in Epidemiology (STROBE) reporting guideline for cohort studies.

### Study Cohort

The Cardiovascular Health Study (CHS) is a prospective, longitudinal cohort study. The study design, rationale, and methods have previously been described in detail.^17,18,19^ In brief, between 1989 and 1990, the study recruited 5201 men and women aged 65 or older in 4 US communities (Sacramento, California; Hagerstown, Maryland; Winston-Salem, North Carolina; and Pittsburgh, Pennsylvania) conducting annual clinical examinations between 1989 and 1999; an additional 687 African American participants were recruited in 1992 to 1993. All participants underwent a medical history, physical examination, laboratory testing, and 12-lead electrocardiography (ECG) at enrollment. Major examination components were repeated during annual follow-up examinations through 1999 and again in 2005. From 1989 to 1999, information about hospitalizations and potential cardiovascular events was collected twice yearly: at annual visits and by telephone 6 months before the annual visits. From 2000 to 2013, participants were contacted every 6 months by telephone, primarily to assess health status for cardiovascular events and physical and cognitive functions.^20^

We included participants with baseline echocardiography, which was performed on the initial cohort recruited between 1989 and 1990. We excluded participants with missing data from the baseline electrocardiogram, and those with a HF diagnosis or borderline or abnormal LVEF at baseline. The additional African American participants recruited from 1992 to 1993 did not have baseline echocardiography performed and were therefore excluded in this study.

### Assessment of Covariates

Covariates were established at the baseline visit. Covariates included study site, age, sex, race, income, body mass index (calculated as weight in kilograms divided by height in meters squared), hypertension, diabetes, coronary artery disease, myocardial infarction, atrial fibrillation (AF), smoking status, alcohol use, and LV mass index from the study echocardiogram. Race was self-reported and categorized as Black or African American, White, or other race (American Indian, Alaska Native, Asian, Pacific Islander, and any race not otherwise specified) because the number of individuals in other racial groups was small. Height and weight were measured by study personnel. Hypertension was defined as either a history of diagnosed hypertension combined with the use of antihypertensive medications, a systolic blood pressure of 140 mm Hg or greater, or a diastolic blood pressure of 90 mm Hg or greater. Diabetes was defined as the use of insulin or hypoglycemic medication at baseline or a fasting glucose level of 126 mg/dL or greater (to convert to millimoles per liter, multiply by 0.0555). Coronary artery disease was defined as angina, previous myocardial infarction, previous coronary artery bypass graft surgery, or previous angioplasty. Myocardial infarction was defined by self-report and confirmed with medical record verification.^18,19^ Prevalent AF was considered present given an extant AF diagnosis, AF on the baseline 12-lead ECG, or on baseline Holter monitoring available from a random subgroup of participants.

### Assessment of Exposure

Twelve-lead resting ECGs were obtained on all participants at baseline using the MAC PC-DT ECG recorder (Marquette Electronics Inc). ECG wave measurements and classification of LBBB followed the guidelines from the Minnesota Code Manual of Electrocardiographic Findings and were overread by a core facility (eTable 1 in Supplement 1).^21^

### Assessment of Outcomes

The methods used to assess cardiovascular events, including HF, have been reported previously.^18,19^ Outcome events were assessed by self-report followed by a confirmational review of the participant’s medical records. The presence of HF was determined by both physician diagnosis and evidence of treatment of HF, which included a current prescription for a diuretic agent and either digitalis or a vasodilator. In addition, the CHS Events Committee reviewed the symptoms, signs, and chest radiograph findings of HF. For each event of incident HF, all medical records 2 weeks before and 30 days after the event were reviewed for LVEF assessments and were considered as systolic dysfunction if the LVEF assessment closest in time was either described qualitatively as below normal or clinically quantified as LVEF less than 45%. Admission with HF with reduced EF (HFrEF) vs HF with preserved EF (HFpEF) was based on only clinical assessments of LVEF at or around the admission.

Study-based echocardiographic methods have been reported previously.^22^ At baseline, M-mode, qualitative 2-dimensional echocardiography, and Doppler imaging were acquired from each participant in the initial cohort recruited in 1989 to 1990. LVEF was qualitatively assessed by a core reading facility from the baseline echocardiogram as normal (≥55%), borderline (≥45% and <55%), or abnormal (<45%).^23,24,25^ These categories follow the American Society of Echocardiography guidelines.^26,27^ LV function was scored in 99% of the initial CHS cohort, with an interreader agreement of 94% and an intrareader agreement of 98% in paired studies.^22^ Echocardiography was repeated after 5 years.^17^ Results from the 5-year echocardiography, overread using the same protocol as baseline, were dichotomized into normal or decreased for the purposes of the current analysis. A decrease in LVEF was defined as a baseline echocardiogram assessed as normal, and a 5-year echocardiography assessed as mildly reduced, moderately reduced, or severely reduced as previously defined.

Deaths were identified during surveillance calls, during scheduling calls for annual clinic visits, or through local daily newspaper obituaries. The events committee adjudicated cause of death using medical records, informant interviews, and death certificates.^18,28^

### Statistical Analysis

Continuous variables with a normal distribution are presented as means with SDs and were compared using the unpaired 2-sample *t* test. Nonnormally distributed continuous variables are presented as medians with IQRs and were compared using the Mann-Whitney *U* test. Categorical variables are presented as frequencies (percentages) and were compared using the χ^2^ test. We used Kaplan-Meier survival analysis to compare the cumulative incidence of HF in patients with and without LBBB over the follow-up period. We performed unadjusted and adjusted Cox proportional hazards models to calculate hazard ratios (HRs) and their 95% CIs for (1) the incidence of HF, (2) the risk of admission for HFrEF and HFpEF (using clinically obtained echocardiograms as previously reported), and (3) the risk of death. In the nonmortality analyses, we used cause-specific models where death was treated as a competing event and censored. The time to event was defined from baseline to the first occurrence of the outcome, death (if not the outcome), or the last date of follow-up, whichever occurred first. Potential confounding variables were chosen based on biological plausibility and the previous literature. These included study site and demographics, comorbidities and lifestyle factors plausibly associated with the risk of HF based on previous literature.^29,30,31,32^ We assessed the proportional hazards assumption using both a visual inspection of a log-minus-log curve and the Schoenfeld test for nonproportional hazards (eFigure in Supplement 1). We performed unadjusted and adjusted logistic regression to calculate the odds of a decrease in LVEF after 5 years using the study echocardiograms performed at baseline and the 5-year visit. We performed 2 sensitivity analyses. First we performed Fine Gray subdistribution hazard models to calculate the HR and the 95% CI for the risk of incident HF, treating death as a competing risk; second, we added adjustment for baseline echocardiographically determined LV mass index in the Cox proportional hazards model assessing time to incident HF. Analysis was performed from February 2018 to October 2024 using Stata version 18 (StataCorp). The threshold for significance was a 2-sided *P* < .05.

## Results

**Figure 1.**
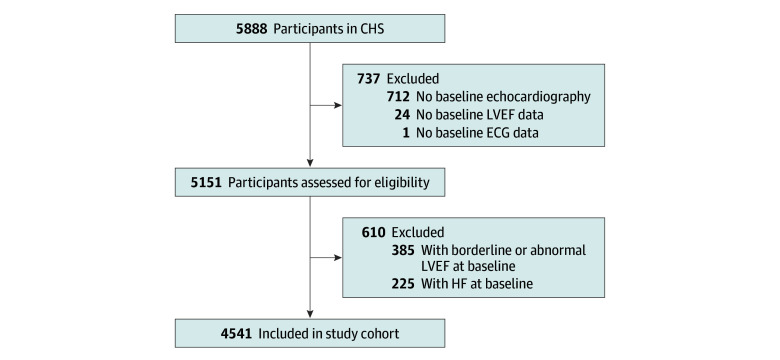
Flow Diagram of the Study Population CHS indicates Cardiovascular Health Study; ECG, electrocardiography; HF, heart failure; LVEF, left ventricular ejection fraction.

The study included 4541 participants (Figure 1). The mean (SD) age was 72.6 (5.5) years, 2697 (59.4%) were female, 44 (1.0%) exhibited a baseline LBBB, and 1321 (29.1%) developed HF over a median (IQR) follow-up of 14.6 (8.4-18.3) years. Baseline characteristics are presented in Table 1. Those with LBBB at baseline were generally older and more likely to have hypertension and diabetes.

**Table 1.** Baseline Characteristics of the Study Population

Characteristics	Participants, No. (%) (N = 4541)		*P* value
	Left bundle branch block (n = 44)	No left bundle branch block (n = 4497)	
Age, mean (SD), y	74.5 (5.7)	72.6 (5.5)	.02
Sex			
Male	14 (31.8)	1830 (40.7)	.23
Female	30 (68.2)	2667 (59.3)	
Race			
Black	3 (6.8)	204 (4.5)	.87
White	41 (93.2)	4271 (95.0)	
Other^a^	0	22 (0.5)	
Income, $			
<12 000	8 (19.5)	949 (22.6)	.89
12 000-34 999	22 (53.7)	2197 (52.2)	
≥35 000	11 (26.8)	1059 (25.2)	
Body mass index, mean (SD)^b^	26.1 (3.6)	26.3 (4.5)	.73
Smoking status			
Never	24 (54.5)	2084 (46.4)	.44
Former	17 (38.6)	1879 (41.8)	
Current	3 (6.8)	531 (11.8)	
No. of alcoholic drinks per week, median (IQR)	0.0 (0.0-0.8)	0.0 (0.0-1.3)	.47
Hypertension	31 (72.1)	2496 (55.6)	.03
Diabetes	11 (25.0)	613 (13.7)	.03
Coronary artery disease	9 (20.5)	686 (15.3)	.34
Myocardial infarction	5 (11.4)	293 (6.5)	.20
Atrial fibrillation	0	90 (2.0)	.34
Left ventricular mass index, mean (SD) g/m^2^	94.6 (17.9)	83.6 (23.1)	.02
Left ventricular internal dimension in diastole, median (IQR), cm	4.9 (4.6-5.5)	4.9 (4.5-5.3)	.36

^a^ Included American Indian, Alaska Native, Asian, Pacific Islander, and any race not otherwise specified.

^b^ Calculated as weight in kilograms divided by height in meters squared.

### Risk of Incident HF

**Figure 2.**
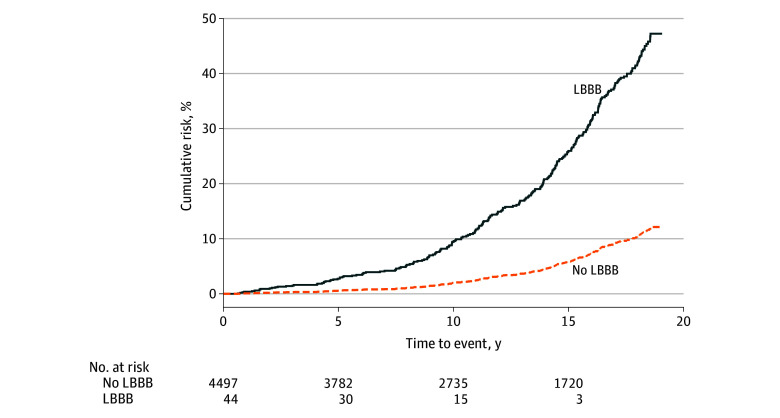
Cumulative Risk of Heart Failure With and Without Left Bundle Branch Block (LBBB)

**Figure 3.**
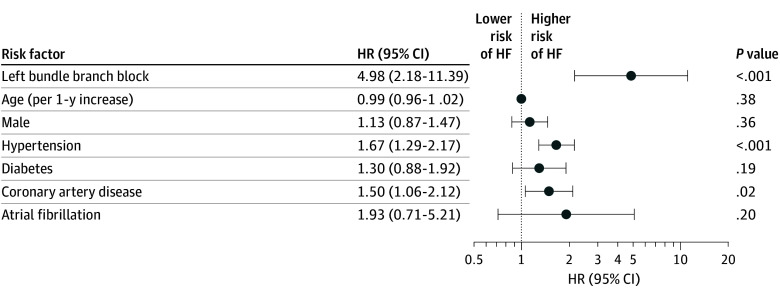
Risk Factors of Heart Failure (HF) HR indicates hazard ratio.

Over a median (IQR) follow-up of 14.6 (8.4-18.3) years, 21 of 44 individuals with LBBB at baseline (47.7%) developed HF compared with 1300 of 4497 individuals without LBBB (28.9%). The unadjusted cumulative risk of HF at the end of follow-up was 48.6% among those with LBBB at baseline vs 12.2% among those without (Figure 2). In the cause-specific Cox proportional hazards model adjusting for potential confounders (age, sex, race, hypertension, diabetes, coronary artery disease, AF, and study site), LBBB at baseline was associated with a higher risk of HF (HR, 4.98; 95% CI, 2.18-11.39; *P* < .001) (Figure 3). The increased risk of incident HF associated with LBBB was larger in magnitude compared with well-established risk factors^33^ of age, sex, hypertension, diabetes, coronary artery disease, and AF (Figure 3). The unadjusted results were overall consistent with the adjusted results (Table 2**)**. The results from the Fine-Gray sensitivity analysis were consistent with these findings (HR, 3.07; 95% CI, 1.33-7.10; *P* = .009). Likewise, the results from the Cox model that included adjusting for baseline LV mass index remained consistent with the primary analysis (HR, 4.99; 95% CI, 1.80-13.79; *P* = .002).

**Table 2.** Unadjusted and Adjusted Results of Baseline Left Bundle Branch Block and the Risk of Main Outcomes

Outcome	Unadjusted HR (95% CI)	*P* value	Adjusted HR (95% CI)^a^	*P* value
Incident heart failure	5.98 (2.65-13.48)	<.001	4.98 (2.18-11.39)	<.001
Heart failure with reduced ejection fraction admission	6.47 (1.58-26.54)	.01	5.98 (1.43-25.00)	.01
Heart failure with preserved ejection fraction admission	3.17 (0.78-12.82)	.11	3.10 (0.76-12.67)	.12
Death	1.75 (1.26-2.44)	.001	1.39 (0.99-1.94)	.05
Left ventricular ejection decrease	OR (95% CI): 4.46 (1.71-11.59)	.002	OR (95% CI): 4.73 (1.70-13.70)	.003

Abbreviations: HR, hazard ratio; OR, odds ratio.

^a^ Adjusted for age, sex, race, hypertension, diabetes, coronary artery disease, atrial fibrillation, and study site.

### Risk of Hospital Admission With HFrEF or HFpEF

In the cohort, a total of 336 participants were admitted to the hospital with HFrEF, and 566 were admitted with HFpEF as confirmed by clinical assessments of LVEF at or around the admission (eTable 2 in Supplement 1). Among 14 participants with baseline LBBB, 11 (78.6%) had either a mild, borderline, or low LVEF upon admission compared with 472 of 864 individuals (54.6%) without LBBB. After adjusting for the same potential confounders as listed previously, LBBB at baseline was associated with a statistically significant higher risk of admission for HFrEF (HR, 5.98; 95% CI, 1.43-25.00; *P* = .01) (Table 2 and eTable 3 in Supplement 1). In the adjusted model, LBBB was not associated with a statistically significantly higher risk of incident HFpEF (HR, 3.10; 95% CI, 0.76-12.67; *P* = .12).

### Risk of LVEF Decrease After 5 Years

Of 3020 individuals with 5-year echocardiograms, 253 (8.4%) experienced a decline in EF. After adjusting for the same potential confounders, LBBB at baseline was associated with a statistically significant higher odds of an LVEF decline (odds ratio, 4.73; 95% CI, 1.70-13.70; *P* = .003) (Table 2 and eTable 4 in Supplement 1).

### Risk of Death

Among those with LBBB at baseline, the risk of death was 82 per 1000 person-years compared with 52 per 1000 person-years among those without. In the unadjusted Cox proportional hazards model, LBBB at baseline was associated with a greater risk of death (HR, 1.75; 95% CI, 1.26-2.44, *P* = .001). After adjusting for the same potential confounders, this association was no longer significant (HR, 1.39; 95% CI, 0.99-1.94; *P* = .05) (Table 2 and eTable 5 in Supplement 1).

## Discussion

In this prospective cohort study of community-dwelling individuals aged 65 years or older with a normal LVEF at baseline, we found that LBBB was associated with an increased risk of incident HF and higher odds of LVEF decline.

Our findings are consistent with previous studies suggesting an association of LBBB with increased risk of HF in asymptomatic individuals.^8,15^ In contrast with past studies that have either assessed HF at limited time points, relied heavily on *International Classification of Diseases, Ninth Revision (ICD-9)* codes for diagnoses and covariate ascertainment, and/or were cross-sectional studies relying only on logistic regression for analyses,^8,15^ this is the first study, to our knowledge, to utilize baseline echocardiography to assure a normal LVEF at baseline and to study such densely phenotyped community-dwelling individuals in a longitudinal fashion.

The increased risk of incident HF associated with LBBB was larger in magnitude compared with other well-established risk factors^33^ of age, sex, hypertension, diabetes, coronary artery disease, and AF, which are prevention targets highlighted in current clinical practice guidelines.^34^ Fitting with the past literature demonstrating improvement in LV systolic dysfunction with biventricular pacing among those with LBBB, our data suggest this HF is primarily due to HFrEF. Indeed, our independent analysis constrained to a comparison of the baseline and 5-year study echocardiograms showed a significant association of baseline LBBB with subsequent LVEF decline. Taken together, these observations suggest that the presence of an LBBB, even in the absence of other evidence of structural heart disease, may be an important independent risk factor for future systolic HF. While the association of LBBB with total mortality lost statistical significance after multivariable adjustment, the point estimate favored a positive association, perhaps pointing to the severity of the presence of this ECG finding and highlighting the seriousness with which such a phenomenon should be considered.

The potential causative role of LBBB in the progression to HF might be explained through several mechanisms. LBBB is characterized by an intraventricular conduction defect, which leads to a dyssynchronous activation and contraction of the LV.^35^ Such inefficient contraction, generally with septal wall contraction followed by lateral wall contraction, can contribute to adverse cardiac remodeling, dilatation, and, ultimately, systolic dysfunction.^12^ Animal models have demonstrated the development of adverse myocardial remodeling caused by LBBB resulting in LBBB-induced cardiomyopathy accompanied by histological changes such as substantial endocardial and myocardial fibrosis, fatty degeneration, vacuolization, and fibrosis within Purkinje fibers.^36^ In humans, LBBB is known to be a frequent finding in patients with HF and dilated cardiomyopathy,^35^ and the reversibility of systolic dysfunction with cardiac resynchronization therapy (such as with biventricular or left bundle area pacing) demonstrates that LBBB can be a cause of cardiomyopathy.^37^

Currently, the finding of asymptomatic LBBB does not prompt further testing or management. Our findings raise the question as to whether early interventions should be considered for these patients to prevent cardiac remodeling, hypertrophy, and, ultimately, HF, or if, perhaps, the presence of an asymptomatic LBBB should trigger regular, protocolized surveillance (such as with serial echocardiograms) to detect the development of systolic dysfunction. Future studies are needed to confirm our findings and improve our understanding of the mechanisms involved, as well as elucidate if and which potential evaluative or preventive interventions might be employed in patients with asymptomatic LBBB. Depending on these findings, future considerations could include whether patients with asymptomatic LBBB should be included as either stage A or stage B HF^38^ (ie, at high risk for HF, but without structural heart disease or symptoms of HF [stage A] or structural heart disease but without signs or symptoms of HF [stage B]). Considering that the risk of progression to HF in this study was comparable with other cardiac risk factors already classified as stage A, the staging of asymptomatic LBBB might provide appropriate and streamlined guidelines on the possible treatment and follow-up of these patients to prevent HF progression. Beyond the management of risk factors as described in the current guideline^38^ (eg, treating hypertension or encouraging smoke cessation), and treating appropriate patients with angiotensin-converting enzyme inhibitors, angiotensin receptor blockers, or β-blockers, these patients might also benefit from a follow-up through longitudinal serial measurement of natriuretic peptides. Finally, these considerations should remain valid whether LBBB is truly causal of HF or simply a marker of future HF risk.

### Limitations

We acknowledge that our study has limitations. Our cohort, derived from CHS, consists of older and primarily White individuals, which may limit the generalizability of our findings to younger populations and other races and ethnicities. The long follow-up period, while a strength for assessing long-term outcomes, may also introduce biases related to changes in diagnostic and therapeutic practices over time.

We cannot exclude the possibility that LBBB is an early manifestation of cardiomyopathy, which later manifests as HF or an LVEF decline (as opposed to the underlying hypothesis that an LBBB causes subsequent systolic dysfunction). However, again given the common success of cardiac resynchronization therapy in many other cohorts,^37,39,40^ LBBB is almost certainly causal in contributing to HF and an LVEF decline in many patients. We assessed LBBB at baseline, rather than as a time-updated variable. This should not have resulted in a type I error (a spurious false positive) but rather, if anything, might suggest an underestimation of the magnitude of the association of LBBB at baseline with increased risk of HF.

The time frame of this study occurred in the absence of modern therapies for HF; those therapies are primarily geared toward treating HF once it manifests and presumably would not have an impact on the outcome of interest for the current investigation, incident HF. However, it is important to acknowledge that drug therapies might have influenced subsequent outcomes such as death.

Although statistically significant associations were observed, the total number of participants with baseline LBBB was relatively low. This may have limited the ability to detect all relevant associations, and future work may combine cohorts to provide sufficient power, particularly for subgroup analyses. Furthermore, because this was an observational study, we cannot exclude residual or unmeasured confounding, making confident causal inferences inappropriate.

## Conclusions

This cohort study of community-dwelling individuals aged 65 years or older with normal baseline systolic function found that baseline LBBB was independently associated with an increased risk of incident HF. These findings suggest that identifying LBBB could be important for identifying high-risk individuals and opens up questions regarding optimal surveillance and potential preventive measures. Furthermore, improving our understanding of the underlying mechanisms might advance future strategies for both the prevention and treatment of HF.

## Notes

Supplement 1. **eTable 1.** Definition of Left Bundle Branch Block **eFigure.** Locally Weighted Scatterplot of Schoenfeld Residuals vs Time to Heart Failure in Years **eTable 2.** Patients Admitted With HFrEF and HFpEF by Baseline Left Bundle Branch Block **eTable 3.** Hazard Ratios for Risk of HFrEF and HFpEF Admission by Covariates **eTable 4.** Odds Ratios for the Decline in Ejection Fraction by Covariates **eTable 5.** Hazard Ratios for Death by Covariates

Supplement 2.
Data Sharing Statement
