# Supplementary material for: Left Bundle Branch Block as a Risk Factor for Heart Failure
Source: JAMA Netw Open. 2025 Aug 7;8(8):e2525801. doi: 10.1001/jamanetworkopen.2025.25801 (PMC12332631; doi:10.1001/jamanetworkopen.2025.25801)
Supplement: Supplement 1. — eTable 1. Definition of Left Bundle Branch Block eFigure. Locally Weighted Scatterplot of Schoenfeld Residuals vs Time to Heart Failure in Years eTable 2. Patients Admitted With HFrEF and HFpEF by Baseline Left Bundle Branch Block eTable 3. Hazard Ratios for Risk of HFrEF and HFpEF Admission by Covariates eTable 4. Odds Ratios for the Decline in Ejection Fraction by Covariates eTable 5. Hazard Ratios for Death by Covariates [file jamanetwopen-e2525801-s001.pdf]

# Supplemental Online Content

Thein A-S, Dixit S, Soliman EZ, et al. Left bundle branch block as a risk factor for heart failure. *JAMA Netw. Open*. 2025;8(8):e2525801.

doi:10.1001/jamanetworkopen.2025.25801

**eTable 1.** Definition of Left Bundle Branch Block

**eFigure.** Locally Weighted Scatterplot of Schoenfeld Residuals vs Time to Heart Failure in Years

**eTable 2.** Patients Admitted With HFrEF and HFpEF by Baseline Left Bundle Branch Block

**eTable 3.** Hazard Ratios for Risk of HFrEF and HFpEF Admission by Covariates

**eTable 4.** Odds Ratios for the Decline in Ejection Fraction by Covariates

**eTable 5.** Hazard Ratios for Death by Covariates

This supplemental material has been provided by the authors to give readers additional information about their work.

© 2025 Thein A-S et al. *JAMA Network Open*.

**eTable 1.** Definition of Left Bundle Branch Block

| | |
|---|---|
| Left bundle branch block | Minnesota Code 7-1-1 or 7-1-2: QRS duration ≥ 0.12 seconds in most beats in leads I, II, III, aVL or aVF, along with an R peak duration ≥ 0.06 seconds in most beats in leads I, II, aVL, V5, or V6; includes those with additional normal QRS complexes of different shape than left bundle branch block pattern. |

© 2025 Thein A-S et al. *JAMA Network Open*.

**eFigure. Locally Weighted Scatterplot of Schoenfeld Residuals vs Time to Heart Failure in Years**

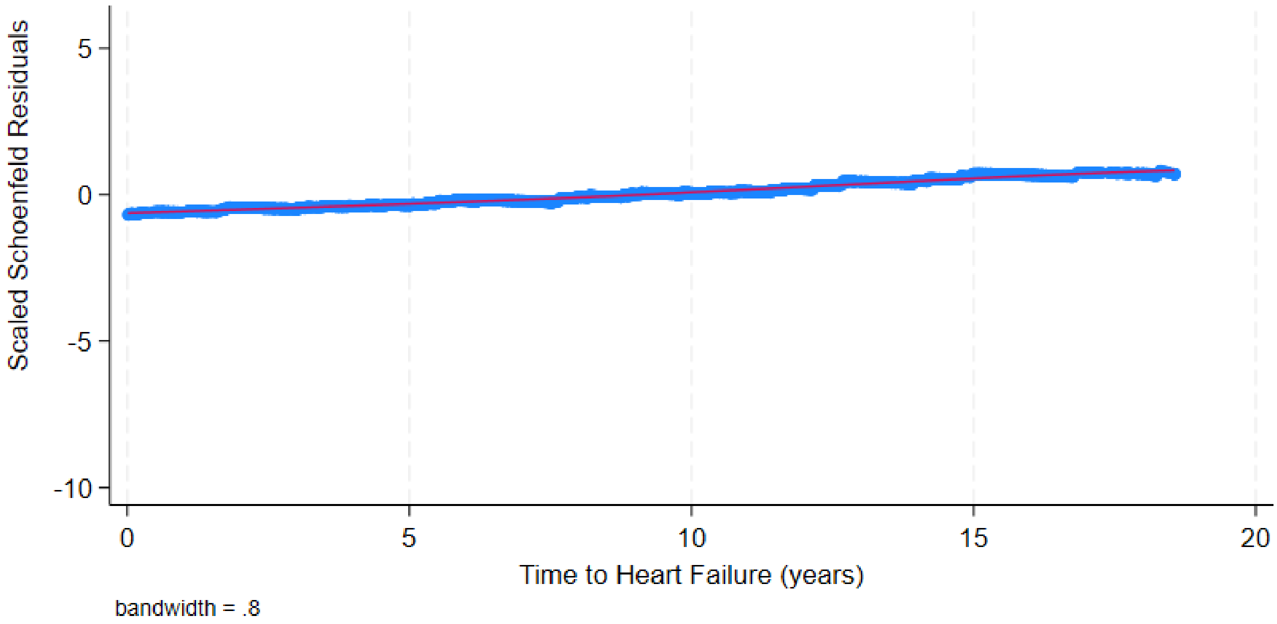


© 2025 Thein A-S et al. *JAMA Network Open*.

**eTable 2.** Percentage of Patients Admitted With HFrEF and HFpEF by Baseline Left Bundle Branch Block

| EF Category | LBBB (%) | No LBBB (%) |
|---|---|---|
| **Normal (≥ 55%)** | 3 (21) | 392 (45) |
| **Mild/borderline (45-55%)** | 4 (29) | 158 (18) |
| **Low (<45%)** | 7 (50) | 314 (36) |

© 2025 Thein A-S et al. *JAMA Network Open*.

## eTable 3. Hazard Ratios for Risk of HFrEF and HFpEF Admission by Covariates

| Outcome | HFrEF (95% CI) | P value | HFpEF (95% CI) | P value |
|---|---|---|---|---|
| **Left bundle branch block** | 5.98 (1.43–25.00) | 0.014 | 3.10 (0.76 – 12.67) | 0.115 |
| **Age (per 1 year increase)** | 1.01 (0.96–1.07) | 0.602 | 0.98 (0.95 – 1.02) | 0.445 |
| **Male** | 2.43 (1.58–3.74) | < 0.001 | 1.22 (0.92 – 1.63) | 0.169 |
| **Race** | | | | |
| **Black** | 2.13 (0.90 – 5.06) | 0.084 | 0.73 (0.30 – 1.80) | 0.492 |
| **Other** | . | . | . | . |
| **Hypertension** | 1.61 (1.04 – 2.50) | 0.034 | 1.35 (1.02 – 1.79) | 0.038 |
| **Diabetes** | 0.52 (0.22 – 1.21) | 0.129 | 1.02 (0.62 – 1.67) | 0.946 |
| **Coronary artery disease** | 1.33 (0.74 – 2.39) | 0.342 | 0.99 (0.64 – 1.54) | 0.965 |
| **Atrial fibrillation** | 1.22 (0.17 – 8.84) | 0.845 | 2.08 (0.66 – 6.59) | 0.212 |

Reference group for race was White.
CI = confidence interval; HFpEF = heart failure with preserved ejection fraction; HFrEF = heart failure with reduced ejection fraction.

© 2025 Thein A-S et al. *JAMA Network Open*.

## eTable 4. Odds Ratios for a Decline in Ejection Fraction by Covariates

| | Odds ratio | Lower CI | Upper CI | P value |
|---|---|---|---|---|
| **Left bundle branch block** | 4.73 | 1.70 | 13.70 | 0.003 |
| **Age (per 1 year increase)** | 1.24 | 0.84 | 1.84 | 0.274 |
| **Male** | 2.43 | 1.85 | 3.10 | < 0.001 |
| **Race** | | | | |
| **Black** | 0.54 | 0.21 | 1.35 | 0.188 |
| **Other** | 1.09 | 0.94 | 9.42 | 0.94 |
| **Hypertension** | 1.18 | 0.90 | 1.55 | 0.238 |
| **Diabetes** | 1.91 | 1.37 | 2.69 | < 0.001 |
| **Coronary artery disease** | 2.62 | 1.94 | 3.54 | < 0.001 |
| **Atrial fibrillation** | 1.16 | 0.45 | 3.01 | 0.764 |

Reference group for race was White.
CI = confidence interval.

© 2025 Thein A-S et al. *JAMA Network Open*.

## eTable 5. Hazard Ratios for Death by Covariates

| | Hazard Ratio | Lower CI | Upper CI | P value |
|---|---|---|---|---|
| **Left bundle branch block** | 1.39 | 0.99 | 1.94 | 0.054 |
| **Age (per 1 year increase)** | 1.12 | 1.11 | 1.12 | < 0.001 |
| **Male** | 1.38 | 1.28 | 1.49 | < 0.001 |
| **Race** | | | | |
| **Black** | 1.07 | 0.91 | 1.27 | 0.402 |
| **Other** | 1.01 | 0.62 | 1.66 | 0.967 |
| **Hypertension** | 1.22 | 1.13 | 1.31 | < 0.001 |
| **Diabetes** | 1.66 | 1.51 | 1.83 | < 0.001 |
| **Coronary artery disease** | 1.37 | 1.25 | 1.51 | < 0.001 |
| **Atrial fibrillation** | 1.52 | 1.22 | 1.92 | < 0.001 |

Reference group for race was White.
CI = confidence interval.

© 2025 Thein A-S et al. *JAMA Network Open*.
