# Supplementary material for: Left Bundle Branch Block as a Risk Factor for Heart Failure
Source: JAMA Netw Open. 2025 Aug 7;8(8):e2525801. doi: 10.1001/jamanetworkopen.2025.25801 (PMC12332631; doi:10.1001/jamanetworkopen.2025.25801)
Supplement: Supplement 2. — Data Sharing Statement [file jamanetwopen-e2525801-s002.pdf]

Data Sharing Statement

Thein. Left Bundle Branch Block as a Risk Factor for Heart Failure. *JAMA Netw Open*. Published August 07, 2025. doi:10.1001/jamanetworkopen.2025.25801

Data
Data available: No

Additional Information
Explanation for why data not available: The data does not belong to the authors. However, the data is available upon reasonable request from the Cardiovascular Health Study.
